# Role of sericin 1 in the immune system of silkworms revealed by transcriptomic and proteomic analyses after gene knockout

**DOI:** 10.1002/2211-5463.13239

**Published:** 2021-07-13

**Authors:** Xiaogang Ye, Shuo Zhao, Meiyu Wu, Jinghua Ruan, Xiaoli Tang, Xiaoxiao Wang, Boxiong Zhong

**Affiliations:** ^1^ College of Animal Sciences Zhejiang University Hangzhou China

**Keywords:** CRISPR/Cas9, proteome, sericin 1, silkworm, transcriptome

## Abstract

The domestic silkworm is a type of lepidopteran insect that feeds on mulberry leaves and has high economic value because of its ability to spin cocoons. Sericin 1 is an important component of silkworm cocoons, accounting for approximately 25% of the material. In this study, CRISPR/Cas9‐mediated gene editing was successfully used to destroy the *sericin 1* gene, and homozygous mutants were obtained after continuous screening. Homozygous mutation resulted in premature termination of the translation of sericin 1 protein at 323 amino acids. Comparative transcriptomic and proteomic analyses of middle silk gland cells from wild‐type individuals and mutants were performed on the fourth day of the fifth instar, and the results suggest that sericin 1 plays an important role in the cellular immune system. In addition, the results suggest that sericin 1 has a synergistic effect with some protease inhibitors and that the secretion of these proteins is strictly regulated. These results will provide new insights into the function and expression pattern of sericin 1 and the mechanism of silk secretion.

## Graphical abstract

CRISPR/Cas9‐mediated gene editing was successfully used to knock out the *sericin 1* gene, and homozygous mutants were obtained. Comparative transcriptomic and proteomic analyses were performed on homozygous mutants and wild‐type individuals to reveal the role of sericin 1 in the defense system.

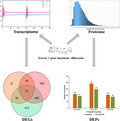

**Abbreviations**
ASG: anterior silk gland
DEGs: differentially expressed genes
DEPs: differentially expressed proteins
FDR: false discovery rate
GO: Gene Ontology
KEGG: Kyoto Encyclopedia of Genes and Genomes
MSG: middle silk gland
PSG: posterior silk gland

The silkworm *Bombyx mori*, domesticated from the Chinese wild silkworm Bombyx mandarina, is an economically important insect because it synthesizes and spins cocoons [1, 2]. The silkworm cocoon is composed of ~ 75% silk fibroin and ~ 25% sericin [3]. Silk fibroin is composed mainly of the fibroin heavy chain (Fib‐H), fibroin light chain (Fib‐L), and 25‐kDa polypeptide proteins (P25) at a molar ratio of 6 : 6 : 1 [4]. Silk fibroin is synthesized and secreted by the posterior silk gland (PSG), while sericin is synthesized and secreted by the middle silk gland (MSG) [5]. In addition to the PSG and MSG, the anterior silk gland (ASG) is responsible for further assembly and secretion of silk [6]. In natural silkworm silk, two parallel bundles of silk fibroin fibers are bonded together by sericin [7].

In the first study of the sericin gene, two kinds of sericin mRNAs, 10 044 bp and 8637 bp in length, were identified from MSGs [8]. Further studies showed that the two sericin mRNAs are transcribed by the *sericin 1* gene [9]. To date, four sericin genes, *sericin 1*, *sericin 2*, *sericin 3*, and *sericin 4*, have been reported in *B. mori*, and the *sericin 4* gene was identified recently [10]. All four sericin genes are located on the 11th linkage group. *Sericin 1* is the most studied sericin gene, and sericin 1 is the main sericin protein in silkworm cocoons [11]. *Sericin 1* is a single‐copy gene with a full length of approximately 24 kb consisting of 9 exons and 8 introns [8, 9]. Four major *sericin 1* mRNAs, 10 044 bp, 8637 bp, 3654 bp, and 2340 bp in length, are produced from the same primary transcript in different sections of the MSG by different splicing methods [9]. Thus far, it has been confirmed that the *sericin 1* gene can produce at least 5 mRNAs of different lengths by alternative splicing [10].

Sericin is a globular protein that irregularly adheres to the periphery of silk fibroin. Sericin contains 18 kinds of amino acids, of which serine, aspartic acid, and glycine account for approximately 64% of the total components [12]. In recent years, a large number of studies have reported that sericin has many biological activities, such as antibacterial [13], antioxidant [14], anticoagulant [15], and anticancer [16] activities and that it promotes cell adhesion and proliferation [17]. Because of these biological characteristics, sericin has been used in many industries, such as the cosmetics [18], biomaterials [19, 20], and food industries [21] and for modification of synthetic fiber materials [22]. In silkworms, sericin binds silk fibroin to form a closed cocoon, which provides protection for the pupae and prevents predation or injury. In addition, sericin can isolate silk fibroin from the external environment so that light, heat, oxygen, and other factors that can easily cause silk fibroin deterioration cannot directly affect the silk fibroin. As the main sericin protein in silkworm cocoons, sericin 1 is considered to be mainly responsible for protecting insects from natural environmental factors.

Clustered regularly interspaced short palindromic repeat (CRISPR)‐Cas9‐mediated gene editing technology, a new and rapidly developing technology, has been widely used in silkworm gene function research [23]. In this study, CRISPR/Cas9‐mediated gene editing technology was successfully applied to knock out the *sericin 1* gene, and corresponding heterozygous and homozygous mutants were obtained. Comparative transcriptomic and proteomic analyses were performed on the MSG cells of mutant and wild‐type individuals on the fourth day of the fifth instar. The results provide insights for further understanding the mechanism of silk secretion and the expression pattern and function of the *sericin 1* gene.

## Materials and methods

### Silkworm strain

*Qiufeng*, a bivoltine silkworm strain maintained by the Silkworm Genetics Laboratory of Zhejiang University, was used in this study. At 3.5 h after oviposition, zygotes were placed in hydrochloric acid for 30 min to eliminate diapause. All silkworms were raised at standard temperature and humidity (25 °C and 80% relative humidity).

### *In* *vitro* preparation of Cas9 mRNA and sgRNA

The SpCas9 expression vector was maintained in our laboratory. The Cas9 plasmids were extracted using a Quick Plasmid Miniprep Kit (Sangon, Shanghai, China). Fifteen micrograms of each Cas9 plasmid was linearized by *XbaI* restriction enzyme digestion (Takara, Kyoto, Japan). The linearized plasmids were then further purified to remove residual RNase A via addition of a solution of 0.5% SDS and 200 µg·mL^−1^ proteinase K and incubation for 30 min at 50 °C; the DNA was then extracted with an equal volume of phenol/chloroform (two times) and precipitated with 2 volumes of ethanol. The purified template DNA was used for mRNA transcription *in vitro* using an SP6 mMESSAGE mMACHINE Kit (Ambion, Waltham, MA, USA) according to the manufacturer’s instructions. The sgRNA template DNA that was synthesized using PCR was purified for mRNA transcription *in vitro* using a T7 mMESSAGE mMACHINE Kit (Ambion) according to the manufacturer’s instructions. The obtained Cas9 mRNAs and sgRNAs were dissolved in RNase‐free PBS solution (pH 7.4), and the final nucleic acid concentrations were measured using a NanoDrop 2000 instrument (Thermo Fisher Scientific, Waltham, MA, USA). The solutions were then aliquoted and stored at −80 °C.

### Microinjection of Cas9 mRNA and sgRNA

Zygote microinjection was performed as previously described [24, 25]. Cas9 mRNAs with a final concentration of 300 ng·μL^−1^ and sgRNAs with a final concentration of 150 ng·μL^−1^ were injected into 1000 zygotes that had eliminated diapause. The injection was completed within 6 h after oviposition. The injected embryos were incubated in an incubator at 25 °C until they hatched.

### Mutagenesis analysis

PCR amplification and cloning of target region sequences were performed to identify genomic mutations. Genomic DNA was extracted using a Genomic DNA Purification Kit (Sangon). Ser1‐F and Ser1‐R primer pairs were used for PCR amplification to produce a 681‐bp target sequence. The PCR conditions were as follows: 94 °C for 5 min; 35 cycles of 94 °C for 30 s, 55 °C for 30 s, and 72 °C for 1 min; and a final extension period of 72 °C for 10 min. The amplified fragments were cloned into the PMD19‐T vector (Takara), and Sanger sequencing was then performed.

### Breeding of homozygous mutants

At the G0 generation, 50 injected embryos were randomly selected to determine the mutation frequency of the G0 generation. The genomic DNA of G0‐generation embryos was extracted for PCR amplification, and the PCR products were cloned and Sanger‐sequenced to identify the mutations. After the injected embryos grew into moths, they were mated with wild‐type moths to produce G1‐generation individuals.

Once the silkworms of the G1 generation had been reared to the third instar, 30 silkworms from each brood were randomly selected for genomic DNA extraction, and then, PCR identification was performed on the obtained genomic DNA to identify the mutant genotypes and mutation frequency. The maintained mutant genotypes were confirmed by analyzing the frameshifts caused by the various mutation types. Genomic DNA from the sloughs was then extracted, and PCR identification was performed to determine the mutant genotypes of the G1‐generation moths. The moths corresponding to the maintained mutant genotypes were mated with wild‐type moths to produce G2‐generation individuals.

Once the silkworms of the G2 generation had grown into moths, the mutant genotypes of the corresponding moths were determined by PCR identification of genomic DNA from the sloughs. Moths with the same mutant genotype were mated in pairs to produce G3‐generation individuals.

Homozygous mutants of the G3 generation were obtained. The theoretical ratio of homozygous mutants, heterozygous mutants, and wild‐type individuals in each brood was 1 : 2 : 1. Similarly, PCR was performed on genomic DNA from the sloughs to identify the mutant genotypes of G3‐generation moths. The homozygous mutant moths were mated with each other to produce G4‐generation individuals. Subsequently, the homozygous mutant population was maintained.

### Tissue sample collection

From the G3 generation, 60 homozygous mutants, heterozygous mutants, and wild‐type individuals were selected on the fourth day of the fifth instar for sample collection. The mutant genotypes of these individuals were determined by PCR using genomic DNA from the head. After the MSGs of each silkworm were dissected in cold 0.9% NaCl solution, the MSGs were put into cold 60% ethanol solution; one end of the MSG was fixed with forceps, and then, the MSG cells were stripped from the MSG with another set forceps and immediately frozen in liquid nitrogen. Each sample was taken from the MSG cells of 20 silkworms, and three biological replicates were prepared. After grinding each sample in liquid nitrogen, some of the MSG powder was used for RNA extraction, and the rest of the powder was used for protein extraction.

### Transcriptomic analysis

RNA sequencing was performed according to a previous study [26]. The library preparations were sequenced on an Illumina HiSeq 2000 platform, and 150‐bp paired‐end reads were generated. The raw data generated by sequencing were filtered using trimmomatic software (v0.36, BGI, Shenzhen, China) to produce clean reads. The clean reads were mapped to the reference silkworm genome database (https://silkdb.bioinfotoolkits.net/base/download/‐1, containing 15 619 entries) using hisat2 software (version v2.1.0) [27] and to the reference gene sequence downloaded from https://silkdb.bioinfotoolkits.net/base/download/‐1 using bowtie2 software (version v2.2.5) [28]. The gene expression levels of each sample were obtained using rsem software (version v1.2.8) [29]. The differentially expressed genes (DEGs) with corrected *P*‐values < 0.05 were identified using degseq software [30].

### Protein extraction and digestion

Protein extraction was performed according to a previously reported method [31]. Briefly, the tissue powder of each sample was resuspended in 300 μL of lysis buffer (containing 7 M urea, 2 M thiourea, 4% 3‐[(3‐cholamidopropyl) dimethylammonio]propanesulfonate (CHAPS), and 40 mm Tris/HCl, pH 8.5) and then treated with 10 mm dithiothreitol (DTT) and 55 mm iodoacetamide. Subsequently, the protein was precipitated with four volumes of cold acetone and redissolved in 200 μL of lysis buffer. After sonication, the supernatant protein solution was obtained by centrifugation. The protein samples were quantified using a Bradford Protein Assay Kit (Tiangen, Beijing, China) according to the manufacturer’s instructions.

A total of 100 μg of protein from each sample was digested with trypsin (sequencing grade, Promega, Madison, WI, USA) at a protein:trypsin ratio of 20 : 1 for 2 h at 37 °C. Before incubation, 0.5 m triethylammonium bicarbonate (TEAB) was added to make the final concentrations of urea and SDS in the protein solution lower than 2 m and 0.1%, respectively.

### iTRAQ analysis

Each trypsin‐digested peptide sample was dissolved in 0.5 m TEAB and combined with the corresponding iTRAQ labeling reagent at room temperature for 2 h. A Shimadzu LC‐20AB liquid‐phase system was used for liquid‐phase separation of the sample, and the separation column was a 5‐μm 4.6 x 250‐mm Gemini C18 column. Combined with the chromatogram, 20 components were obtained and then freeze‐dried.

Each freeze‐dried peptide sample was redissolved with mobile phase A (2% acetonitrile, 0.1% formic acid) and centrifuged at 20 000 × ***g*** for 10 min, and the supernatant was used for injection. Separation was performed using a Thermo UltiMate 3000 UHPLC system (Thermo Fisher Scientific). The samples were first enriched and desalted in a trap column and then separated in series with a self‐packed C18 column (75‐μm internal diameter, 3‐μm column size, 25‐cm column length) at a flow rate of 300 nL·min^−1^ through the following effective gradient: 5% mobile phase B (98% acetonitrile, 0.1% formic acid) for 5 min, 5%–25% mobile phase B over 40 min, 25%–35% mobile phase B over 5 min, 35%–80% mobile phase B over 2 min, 80% mobile phase B for 2 min, and 5% mobile phase B for 6 min.

The peptides separated by liquid‐phase chromatography were ionized by a nanoESI source and then entered a Q Exactive HF X tandem mass spectrometer (Thermo Fisher Scientific) for detection in data‐dependent acquisition (DDA) mode. The main parameters were set as follows: ion source voltage, 1.9 kV; MS1 scanning range, 350–1500 m/z; resolution, 60 000; MS2 starting m/z, fixed at 100; and resolution, 15 000. The top 20 parent ions with a peak intensity exceeding 10 000 and a charge of 2+ to 6+ were screened for MS2 fragmentation. The ion fragmentation mode was HCD. The dynamic exclusion time was set to 30 s. The AGC was set to MS1 3E6 and MS2 1E5.

### Protein identification and quantification

The MS/MS raw data were subjected to a search for peptide/protein identification and quantification using Mascot Daemon software (version 2.3.02, Matrix Science, London, UK). The searches were executed against an open silkworm proteome database downloaded from the Silkworm Genome Database website (https://silkdb.bioinfotoolkits.net/base/download/‐1). The parameters for the Mascot search were set as follows: type of search, MS/MS ion search; enzyme, trypsin; fragment mass tolerance, 0.05 Da; mass values, monoisotopic; variable modifications, oxidation, deamidated; peptide mass tolerance, 20 ppm; and fixed modifications, carbamidomethyl. Each protein identification was supported by at least one unique peptide identification. To improve the reliability of the identified peptides, the PSMs were prefiltered at an false discovery rate (FDR) of 1%. To control the rate of false positives at the protein level, the proteins were filtered at an FDR of 1%, based on the ‘picked’ protein FDR strategy [32]. The labeled peptides with isobaric tags were quantitatively analyzed using IQuant software [33] with the integrated Mascot Percolator algorithm [34]. Proteins meeting the two criteria of a *P*‐value < 0.05 and a fold change ≥ 1.2 or ≤ 0.83 were considered significantly differentially expressed between the two samples.

### Bioinformatic analysis

Gene Ontology (GO) analyses were performed according to http://en.wikipedia.org/wiki/Gene_Ontology. GO analyses of the DEGs and differentially expressed proteins (DEPs) were performed with the phyper function in R software. GO terms with corrected *P*‐values ≤ 0.05 were considered significantly enriched. Kyoto Encyclopedia of Genes and Genomes (KEGG) pathway analyses of the DEGs and DEPs were performed according to http://www.genome.jp/kegg/pathway.html. KEGG pathways with corrected *P*‐values ≤ 0.05 were considered significantly enriched.

## Results

### Production of *sericin 1* knockout mutants

**Fig. 1:**
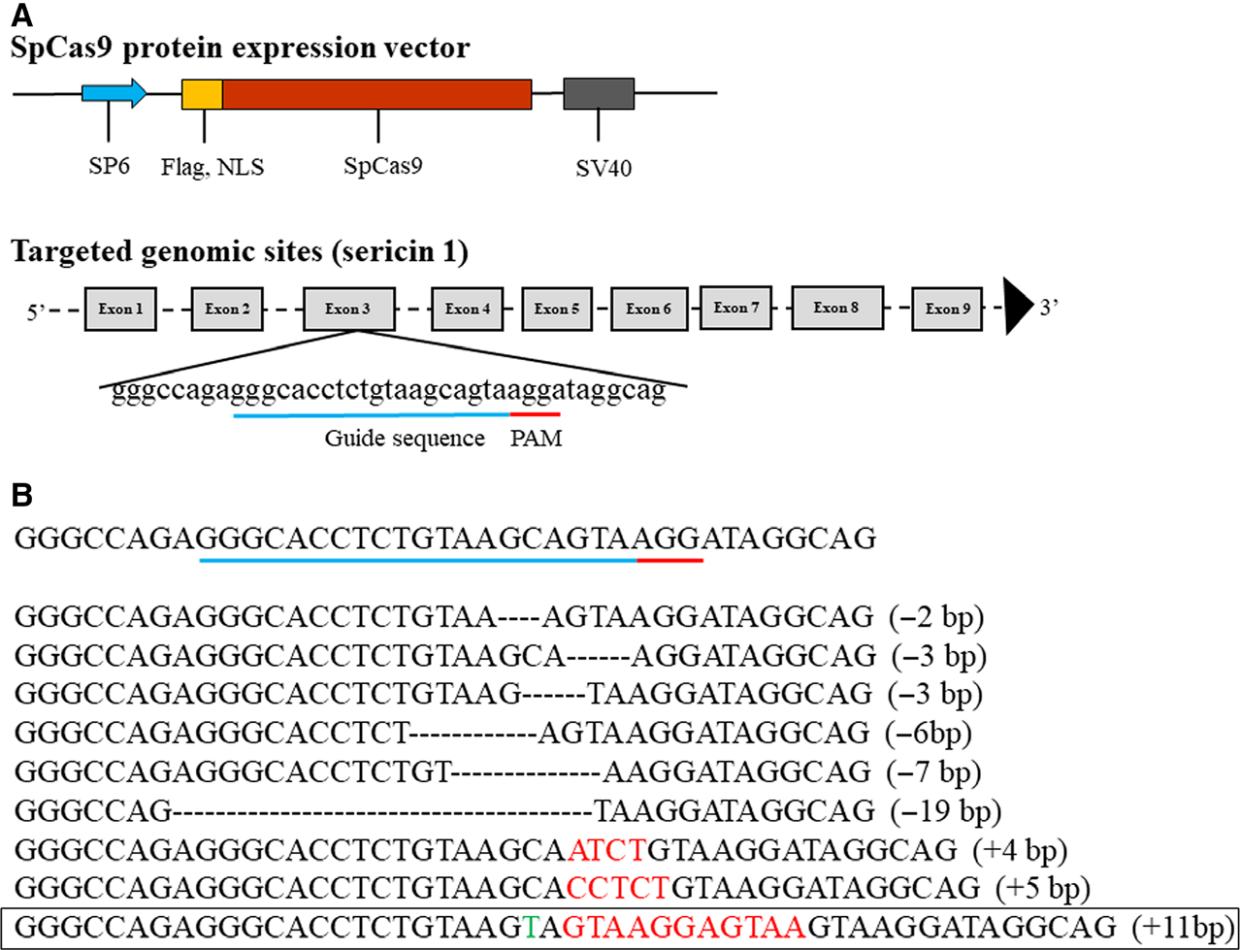
Construction of the CRISPR/Cas9 system targeting *sericin 1*. (A) Schematic overview of the SpCas9 protein expression vector and targeting site. The dashes represent the *sericin 1* genome locus. SP6, SP6 promoter; NLS, nuclear localization sequence; SV40, poly(a) sequence; PAM, protospacer‐adjacent motif; gray boxes, exons. (B) Identified mutant sequence. Deletions and insertions are indicated by dashes and red letters, respectively. Base substitutions are indicated with green letters. The base number of the indel is shown in brackets. The black rectangle indicates the type of mutation used in this experiment.

**Fig. 2:**
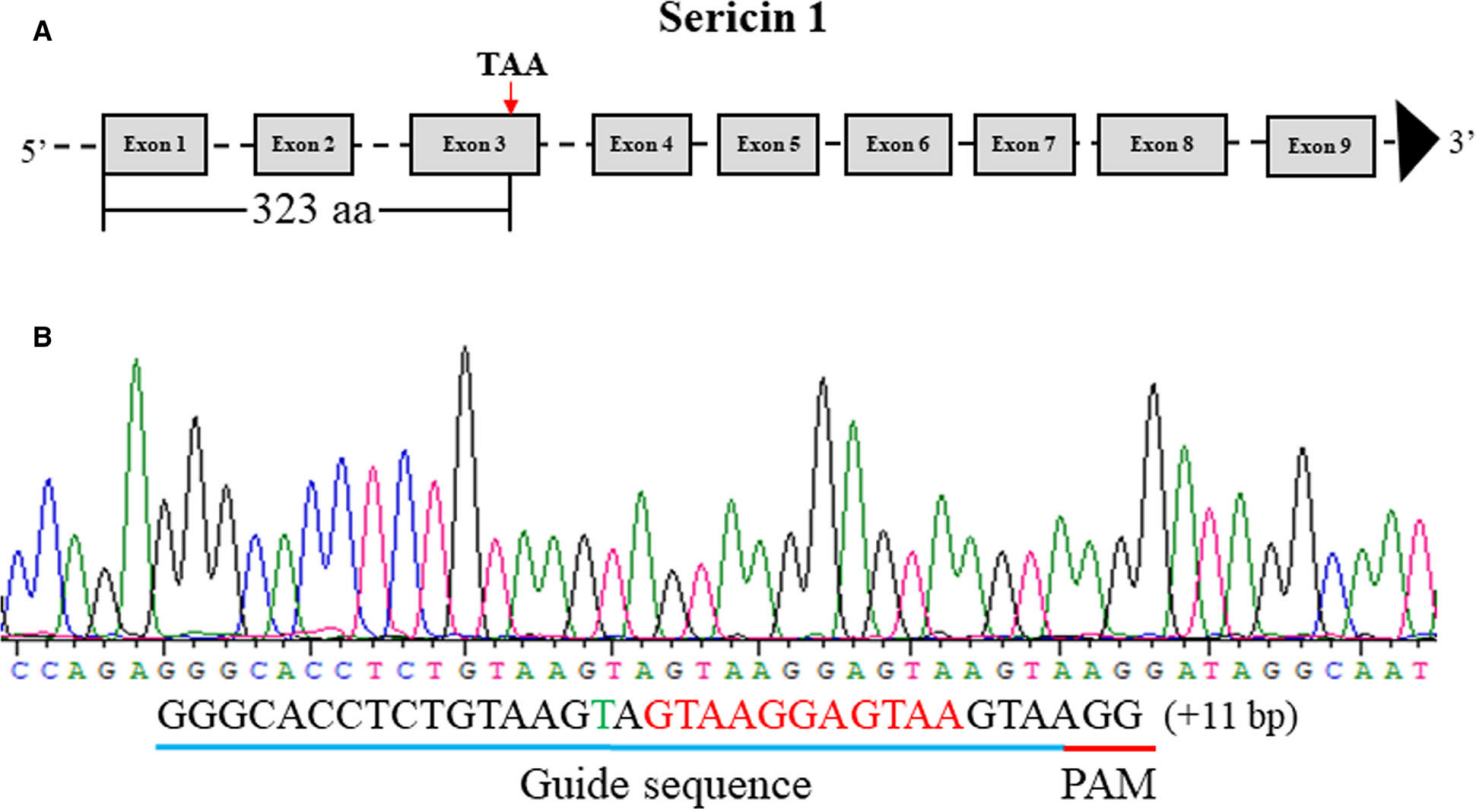
Schematic overview of premature termination of sericin 1 translation and the sequencing chromatogram of homozygous mutants. (A) Premature termination of sericin 1 translation. TAA, stop codon. The red arrow indicates the location of premature termination of translation. (B) Sequencing chromatogram of homozygous mutants. Insertions are indicated with red letters. Base substitutions are indicated with green letters. PAM, protospacer‐adjacent motif.

**Fig. 3:**
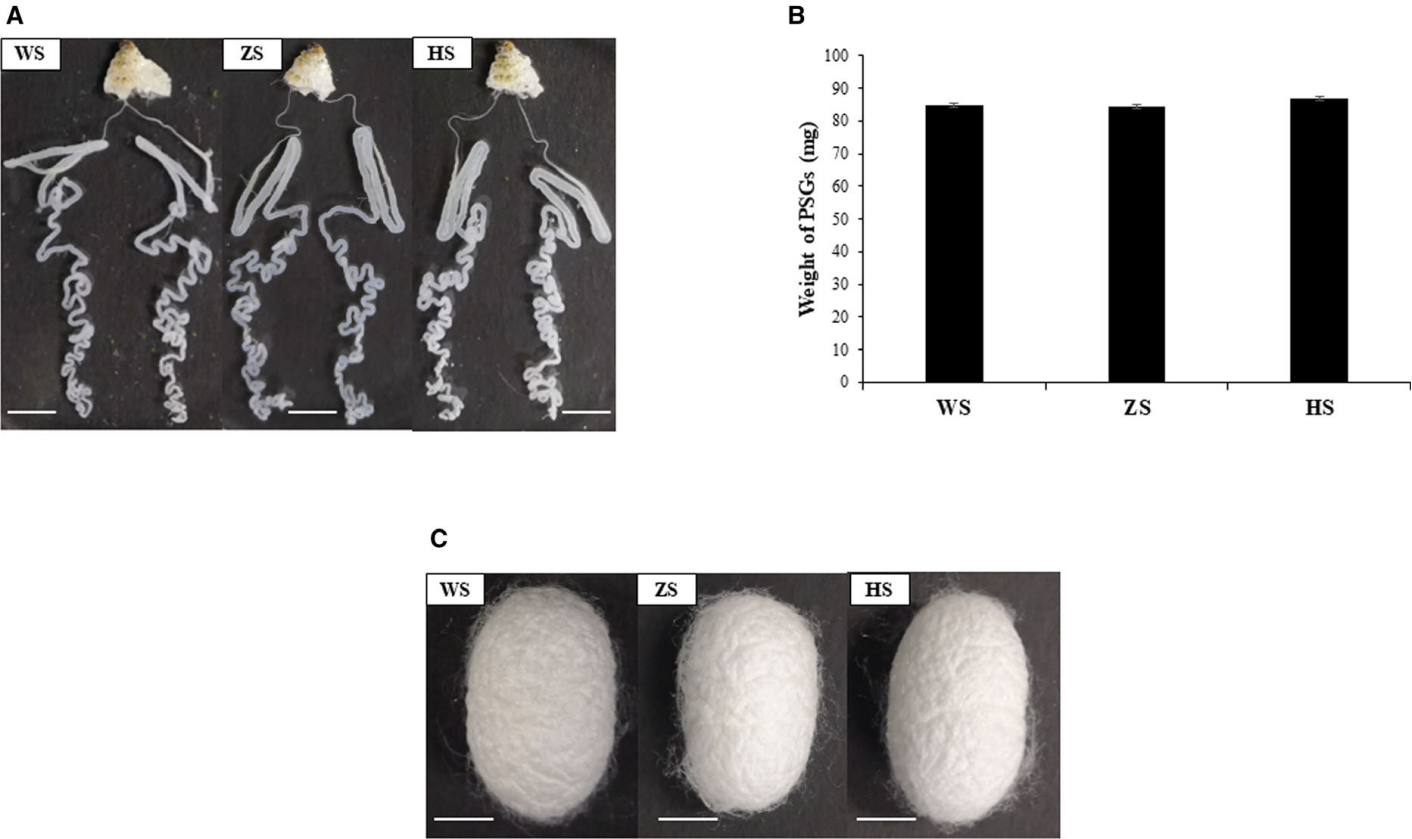
Phenotypes of mutants. (A) Silk glands of WS, ZS, and HS individuals on the third day of fifth instar. (B) PSG weights of the WS, ZS, and HS groups on the fourth day of the fifth instar. Thirty PSGs were collected from individuals of each genotype, and the difference in the PSG weight was analyzed by Student's *t*‐test. Error bars indicate standard deviation. (C) Cocoon shells of WS, ZS, and HS individuals. All the scale bars represent 1 cm.

The *sericin 1* gene has 9 exons, and the first and second exons encode signal peptide sequences [9]. In this study, an sgRNA targeting the third exon of the *sericin 1* gene was constructed (Fig. 1A, Table S1). Cas9 mRNA and sgRNA that were synthesized *in vitro* were premixed and injected into 1000 silkworm embryos within 6 h of oviposition. After 72 h of normal culture in an incubator, 50 embryos were randomly selected for genomic DNA preparation; the target region of the *sericin 1* gene (681 bp) was then amplified by PCR using the Ser1‐F and Ser1‐R primer pair, and the product was used for Sanger sequencing to identify mutations. The Sanger sequencing results showed that the genomes of 46 (92%) embryos had been modified (Table S2). In the G0 generation, 312 ant silkworms hatched, and 210 moths were obtained. The mating of G0‐generation moths with wild‐type moths produced 184 broods of the G1 generation. In the G1 generation, 50 broods of silkworms were randomly selected from 184 broods and raised under standard conditions. When the silkworms were reared to the third instar, 30 larvae from each brood were randomly selected for genomic DNA preparation; the target region of the *sericin 1* gene was then amplified by PCR, and the product was cloned into the T‐vector, after which Sanger sequencing was performed. The Sanger sequencing results suggested that 42 of 50 broods experienced significant genomic modification; thus, the corresponding G1‐generation mutation frequency was 84%, which was not significantly different from that of the G0 generation (Table S2). In addition, the mutation sequences were analyzed. The results showed that the insertion mutation occurred at the cleavage site, the largest insertion was 11 bp, the deletion mutation mainly occurred upstream of the cleavage site, and the largest deletion was 19 bp (Fig. 1B). After screening, an 11‐bp insertion mutation type was selected, and homozygous mutants were obtained and maintained. This mutation resulted in a frameshift mutation and a premature stop codon at 323 aa (Figs 2A and S1). Through directional breeding, homozygous mutants with 11‐bp insertions were obtained in the G3 generation. The homozygous mutation was confirmed with a sequencing chromatogram generated by PCR identification of genomic DNA from the heads of silkworms (Fig. 2B). In the G3 generation, the silk glands of wild‐type individuals, heterozygous mutants, and homozygous mutants on the third day of the fifth instar were dissected for phenotypic observation. For convenience of description, wild‐type individuals, heterozygous mutants, and homozygous mutants are abbreviated as WS, ZS, and HS individuals, respectively. The results showed that there was no significant difference in MSG phenotype among WS, ZS, and HS individuals (Fig. 3A). Previous studies have shown that knocking out the *fibroin heavy chain* gene causes premature degeneration of PSGs [6]. Silk is composed mainly of sericin and silk fibroin, and knockout of the *sericin 1* gene may affect the expression levels of fibroin‐related genes, so the phenotype of PSGs was investigated. The results showed that there was no significant difference in PSG phenotype among WS, ZS, and HS individuals (Fig. 3A). Furthermore, the PSGs of thirty WS, ZS, and HS individuals were dissected and weighed on the fourth day of the fifth instar, and the results showed no significant differences among them, consistent with the phenotypic results (Fig. 3B). Subsequently, the cocoon shell phenotypes of WS, ZS, and HS individuals were observed, and no differences were found among them (Fig. 3C).

### Identification of DEGs by comparative transcriptomic analysis of MSG cells

Nine samples of MSG cells from WS, ZS, and HS individuals on the fourth day of the fifth instar were subjected to RNA sequencing using an Illumina HiSeq 2000 platform, and an average of 6.39 G of data was produced per sample (Table S3). In total, 155.12 M, 151.86 M, and 146.94 M raw reads were obtained from the WS‐MSGs, ZS‐MSGs, and HS‐MSGs. After filtering, 128.15 M, 128.87 M, and 126.57 M clean reads were obtained from the WS‐MSGs, ZS‐MSGs, and HS‐MSGs (Table S3). Values of Q20 > 97% and values of Q30 > 94% in all quality scores suggested a high confidence level for the RNA sequencing performed in this study (Table S3). Subsequently, the clean reads were mapped to the reference silkworm genome. The mapping rate was 73.45%–75.59%, and the unique mapping rate was 42.44%–48.9% (Table S4). The clean reads were mapped to the reference silkworm gene sequences, the mapping rate was 60.74%–70.48%, and the unique mapping rate was 37.66%–46.9% (Table S5). In the end, a total of 12 404 assembled transcripts were identified by RNA sequencing (Table S6).

**Fig. 4:**
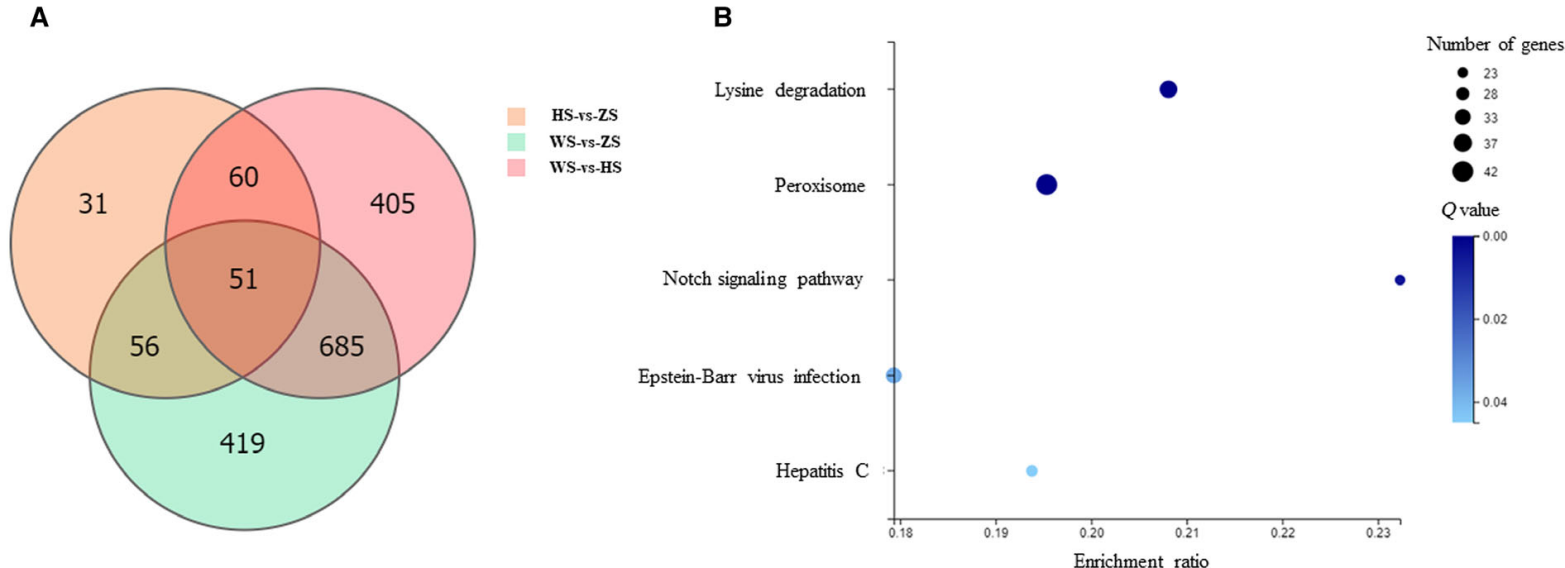
DEGs identified in transcriptome analysis and the results of KEGG enrichment analysis of the DEGs. (A) Venn diagram of the DEGs identified from each comparison. (B) KEGG enrichment analysis of the DEGs identified from the comparison of HS‐MSGs and WS‐MSGs. A corrected *P*‐value ≤ 0.05 was considered to indicate significant enrichment.

The gene expression levels in each sample were calculated based on the fragments per kb per million reads (FPKM) values. The DEGs were identified based on the negative binomial distribution using DEGseq software [30]. In total, 1211 DEGs (577 upregulated and 634 downregulated) were identified from the comparison of the ZS‐MSGs with the WS‐MSGs (Table S7); 1201 DEGs (538 upregulated and 663 downregulated) were identified from the comparison of the HS‐MSGs with the WS‐MSGs (Table S8); and 198 DEGs (64 upregulated and 134 downregulated) were identified from the comparison of the ZS‐MSGs with the HS‐MSGs (Table S9). In addition, a Venn diagram was generated to show the shared DEGs between the comparison groups. In total, 111 shared DEGs were identified from both the comparison of the HS‐MSGs and WS‐MSGs and the comparison of the ZS‐MSGs and HS‐MSGs; 736 shared DEGs were identified from both the comparison of the HS‐MSGs and WS‐MSGs and the comparison of the ZS‐MSGs and WS‐MSGs; and 107 shared DEGs were identified from both the comparison of the ZS‐MSGs and WS‐MSGs and the comparison of the ZS‐MSGs and HS‐MSGs (Fig. 4A).

### Bioinformatic analysis of the DEGs

Previous studies have reported that *fibroin heavy chain* gene knockout heterozygous mutants and wild‐type individuals have no significant differences in either phenotype or gene expression levels, while there are significant differences between homozygous mutants and wild‐type individuals [6]. Therefore, the DEGs identified from the comparison of the HS‐MSGs with the WS‐MSGs were screened for bioinformatic analysis. First, the expression level of the *sericin 1* gene was investigated. In WS‐MSGs, the average FPKM value of the *sericin 1* gene from three biological replicates was 69 711, whereas in HS‐MSGs, the average FPKM value of the *sericin 1* gene from three biological replicates was 22 910 (Table S6). Compared with that in WS‐MSGs, the expression level of the *sericin 1* gene was more than 3 times lower in HS‐MSGs, which further indicated that the *sericin 1* gene was successfully knocked out. However, compared with those of other transcripts, the expression levels of the *sericin 1* gene in HS‐MSGs remained very high, for two possible reasons. First, homozygous mutations produce frameshift mutations and premature termination at 323 aa; however, transcription may still occur under the action of an endogenous promoter. It can be inferred that abnormal transcripts can still be detected. Second, the *sericin 1* gene can produce at least five mRNAs by alternative splicing, and the third exon is a selectable exon [10], so it can be inferred that the mutation does not affect the normal formation of transcripts without the third exon. Next, the expression levels of the *sericin 2* and *sericin 3* genes were investigated. Four sericin proteins, sericin 1, sericin 2, sericin 3, and sericin 4, have been reported in silkworms, all of which are secreted by MSG cells [10]. Compared with those in WS‐MSGs, the average FPKM values of the *sericin 2* gene were approximately 1.7 times higher in HS‐MSGs, and the average FPKM values of the *sericin 3* gene were also slightly higher (Table S6), suggesting that there may be common transcriptional regulatory factors among sericin genes. MSG cells compensate for the drastic decrease in *sericin 1* expression by increasing the expression levels of *sericin 2* and *sericin 3*.

Gene Ontology analysis was performed on the DEGs identified from the comparison of HS‐MSGs and WS‐MSGs, and GO terms with corrected *P*‐values ≤ 0.05 were considered significantly enriched. The significant enrichment results showed that the DEGs were significantly enriched for 14 GO items, 10 of which were related to catalytic activity, such as DNA polymerase activity, endonuclease activity, nuclease activity, and hydrolase activity (Fig. S2). Furthermore, KEGG pathway analysis was performed on the DEGs identified from the comparison of HS‐MSGs and WS‐MSGs, and pathways with corrected *P*‐values ≤ 0.05 were considered significantly enriched. The significant enrichment results showed that the DEGs were significantly enriched only for five pathway terms: lysine degradation, peroxisome, the notch signaling pathway, Epstein–Barr virus infection, and hepatitis C (Fig. 4B, Table S10). It is notably that the DEGs were significantly enriched in the Epstein–Barr virus infection and hepatitis C pathways, as this result indicates that downregulation of *sericin 1* gene expression has a great impact on the immune function and homeostasis of MSG cells. In silkworm cocoons, sericin, composed of a group of coated proteins, is coated on fibroin to form a closed space that helps the silkworms resist attacks from natural enemies and microbial infections. It cannot be ignored that sericin itself has the ability to resist microbial infection, which has been confirmed by many studies [13, 35]. Sericin has been used in biomedical materials because it can resist microbial infection [36]. It can be inferred that sericin 1 may not only be a secretory protein but also participate in the cellular immune response in MSGs. To further confirm that downregulation of *sericin 1* gene expression affects the cellular immune response, the DEGs associated with the Epstein–Barr virus infection and hepatitis C pathways were analyzed; these included *cytochrome c* (*Cytc*), *inhibitor of nuclear factor kappa‐B kinase* (*IKK*), *RAC serine/threonine protein kinase* (*RAC‐PK*), *thioredoxin domain‐containing protein* (*TXNDC*), *calreticulin*, *scavenger receptor class B member 4* (*SCARB4*), *a*nd *lysosome membrane protein 2* (*SCARB1*). Cytc activates the apoptosis signal transduction pathway to resist virus infection [37]. IKK, as a protease inhibitor, regulates the immune response [38]. RAC‐PK, as a serine/threonine protein kinase, is activated by cellular stress [39]. TXNDC, as a potent antioxidant, shows significant responses to immune stimuli [40]. Calreticulin serves as a marker for phagocytosis of apoptotic cells, and phagocytosis is a central mechanism in the defense against infectious agents [41]. SCARB1 and SCARB4 play an important role in innate immunity, and they can recognize several different microbial ligands from bacteria and fungi, including lipoteichoic acid, CpG DNA, and bacterial lipopolysaccharide [42, 43]. It is worth noting that the abovementioned genes were significantly upregulated in HS‐MSGs compared with WS‐MSGs, which indicated that cells may respond to downregulation of *sericin 1* gene expression by upregulating the expression of some genes related to the immune response to achieve a new cell homeostasis. These results confirm that sericin 1 is not only a secreted protein but also directly related to the cellular immune process, which is strictly regulated by relevant transcriptional regulatory factors.

In silkworm cocoons, in addition to sericins and fibroins, there are a variety of protease inhibitors that can effectively inhibit the protease activity of fungi and bacteria [11, 44]. Comparative proteomic analysis of domestic silkworm and wild silkworm cocoons has shown that many protease inhibitors are significantly upregulated in wild silkworm cocoons compared with domestic silkworm cocoons [45]. In addition, the content of sericin in wild silkworm cocoons is significantly higher than that in domestic silkworm cocoons [45]. These results suggest that protease inhibitors and sericin play a synergistic role in the cocoon shell against microbial infection, thus protecting the pupa within the cocoon. It can be inferred that the decrease in *sericin 1* gene expression might affect the expression levels of genes encoding protease inhibitors, so the expression levels of genes encoding protease inhibitors were then investigated. The results showed that 16 genes encoding protease inhibitors were significantly upregulated, including *fungal protease inhibitor F* (*FPI‐F*), *zonadhesin*, and *BCP inhibitor* (*BCPI*), in HS‐MSGs compared with WS‐MSGs (Table 1). Zonadhesin can significantly inhibit the conidial germination of *Beauveria bassiana* and significantly increase the survival rate of silkworms [46]. In addition, only two genes encoding protease inhibitors were downregulated in HS‐MSGs compared to WS‐MSGs: *serine protease inhibitor 15* and *serine protease inhibitor 17* (Table 1). Notably, all the proteins corresponding to the genes encoding protease inhibitors that were upregulated in HS‐MSGs have been detected in the cocoon [45], suggesting that the secretion of protease inhibitors is strictly regulated rather than occurring along with liquid silk secretion from the silk gland. These results suggest that the cells compensate for drastic downregulation of *sericin 1* gene expression by upregulating genes encoding protease inhibitors to ensure cell homeostasis. They also suggest that there is an associated regulatory pathway between the *sericin 1* gene and the genes encoding protease inhibitors that is also associated with the cellular immune response.

**Table 1:** DEGs encoding protease inhibitors identified from the comparison of the HS‐MSGs with the WS‐MSGs.

Gene ID	WS FPKM	HS FPKM	log2(HS/WS)	Q value (WS‐vs‐HS)	*P* value (WS‐vs‐HS)	NCBInr Description
Upregulated						
BMSK0001557	291.353	1606.483	1.91233691	1.66E‐93	1.41E‐96	AKJ54535.1|1.2e‐53|fungal protease inhibitor [Bombyx mori]
BMSK0001558	189.56	636.593	1.234707224	2.07E‐16	2.16E‐18	XP_021202481.1|6.4e‐49|fungal protease inhibitor F‐like [Bombyx mori]
BMSK0001601	63.83	145.806	0.68432769	6.11E‐06	1.86E‐07	XP_004926666.2|7.2e‐91|alaserpin isoform X1 [Bombyx mori]
BMSK0003466	1494.023	2564.443	0.282559385	0.031099164	0.002847329	XP_004925684.1|9.2e‐159|SCO‐spondin [Bombyx mori]
BMSK0009587	1731.753	4600.003	0.95200668	3.73E‐21	2.88E‐23	XP_012551854.1|6.3e‐104|phosphatidylethanolamine binding protein isoform X1 [Bombyx mori]
BMSK0010135	71.233	144.9	0.522205733	0.002822299	0.000158868	NP_001037057.1|6.6e‐46|BCP inhibitor precursor [Bombyx mori]
BMSK0010509	1.176	3.933	1.188447277	0.000416484	1.88E‐05	XP_004928169.1|0.0e+00|inhibitor of nuclear factor kappa‐B kinase subunit beta [Bombyx mori]
BMSK0012818	2001.73	7199.81	1.356878373	5.65E‐45	1.48E‐47	XP_012546580.1|4.2e‐107|serine protease inhibitor 28 isoform X1 [Bombyx mori]
BMSK0012819	48.366	177.226	1.365036388	2.86E‐22	1.99E‐24	XP_021204235.1|2.8e‐87|zonadhesin [Bombyx mori]
BMSK0012821	22.536	181.046	2.488499146	3.01E‐63	5.36E‐66	XP_021204235.1|1.0e‐89|zonadhesin [Bombyx mori]
BMSK0012822	709.503	3639.426	1.869100117	7.65E‐82	9.73E‐85	XP_021204235.1|0.0e+00|zonadhesin [Bombyx mori]
BMSK0012982	673.133	1591.996	0.745443729	1.04E‐09	1.89E‐11	XP_012544229.1|4.2e‐91|zonadhesin isoform X1 [Bombyx mori]
BMSK0012983	666.643	1810.623	0.911002139	5.09E‐10	8.76E‐12	XP_021202413.1|3.6e‐42|inducible metalloproteinase inhibitor protein‐like isoform X1 [Bombyx mori]
BMSK0012984	12.57	72.666	1.83671194	5.38E‐05	1.98E‐06	XP_012544229.1|1.5e‐39|zonadhesin isoform X1 [Bombyx mori]
BMSK0012986	176.85	966.036	1.76838498	6.38E‐06	1.99E‐07	XP_012544229.1|8.3e‐38|zonadhesin isoform X1 [Bombyx mori]
BMSK0015993	32.636	58.8	0.349636315	0.046785389	0.004686042	NP_001037090.1|6.0e‐232|serine protease inhibitor 4 precursor [Bombyx mori]
Downregulated						
BMSK0001599	10.236	7.426	−1.265750981	5.69E‐10	9.94E‐12	NP_001139707.1|1.9e‐222|serine protease inhibitor 15 precursor [Bombyx mori]
BMSK0001600	12.18	1.023	−3.852308358	1.53E‐30	7.01E‐33	NP_001139710.1|7.4e‐219|serine protease inhibitor 17 precursor [Bombyx mori]

### Identification of DEPs by comparative proteomic analysis of MSG cells

**Fig. 5:**
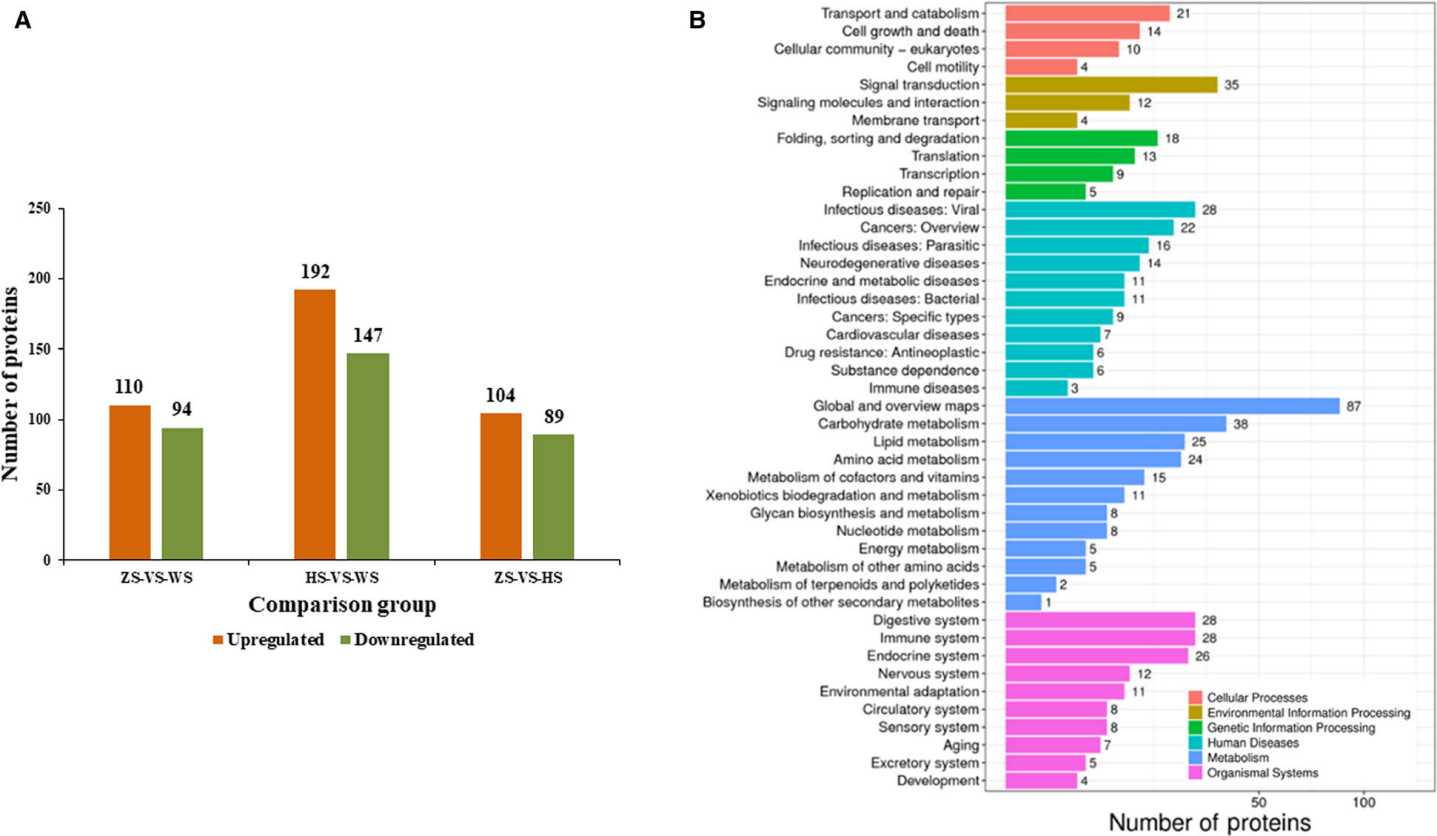
DEPs identified in proteomic analysis and the results of KEGG enrichment analysis of the DEPs. (A) Bar chart of the DEPs identified from each comparison. (B) KEGG enrichment analysis of the DEPs identified from the comparison of HS‐MSGs and WS‐MSGs.

Six samples of MSG cells from WS, ZS, and HS individuals on the fourth day of the fifth instar were analyzed by iTRAQ‐based proteomics. A total of 905 177 secondary spectra were obtained from six samples. A total of 30 482 peptides and 4548 proteins were identified after filtration under a PSM‐level FDR of 1% and a protein‐level FDR of 1% (Table S11). Of the 4548 identified proteins, 3458 (76%) had two or more unique peptides (Fig. S3). The identified proteins were quantified using IQuant software [33]. The DEPs were determined using two criteria: a *P*‐value < 0.05, a fold change ≥ 1.2 or ≤ 0.83. In total, 204 DEPs (110 upregulated and 94 downregulated) were identified from the comparison of the ZS‐MSGs with the WS‐MSGs (Fig. 5A, Table S12); 339 DEPs (192 upregulated and 147 downregulated) were identified from the comparison of the HS‐MSGs with the WS‐MSGs (Fig. 5A, Table S13); and 193 DEPs (104 upregulated and 89 downregulated) were identified from the comparison of the ZS‐MSGs with the HS‐MSGs (Fig. 5A, Table S14).

### Bioinformatic analysis of the DEPs

Similar to the procedure for the transcriptomic analysis, the DEPs identified from the comparison of the HS‐MSGs with the WS‐MSGs were screened for bioinformatic analysis. First, the expression level of sericin 1 was investigated. The results showed that the expression level of sericin 1 in HS‐MSGs was nearly 3 times lower than that in WS‐MSGs, which further indicated that the *sericin 1* gene was successfully knocked out (Table S11). As revealed in the transcriptomic analysis, sericin 1 expression was downregulated in HS‐MSGs, but it was still at a relatively high level because of the contribution of two proteins: the abnormal protein caused by mutation and the sericin 1 protein without exon 3 caused by alternative splicing. Second, the expression levels of sericin 2 and sericin 3 were investigated. Compared with that in WL‐PSGs, the expression level of sericin 2 in HL‐PSGs was upregulated by 1.19‐fold, which further indicated that the cells responded to the loss of sericin 1 by increasing the expression of sericin 2 (Table S11). However, sericin 3 was not detected in proteomic tests, possibly because of low sericin 3 abundance or the limitations of mass spectrometry.

A GO analysis of the DEPs identified from the comparison of HS‐MSGs and WS‐MSGs was performed (Fig. S4). In the molecular function category, catalytic activity and binding were the most represented terms, whereas in the cellular component category, cell part and cell were the most represented terms. Moreover, in the biological process category, metabolic process and cellular process were the most represented terms. Furthermore, a KEGG pathway analysis of the DEPs identified from the comparison of HS‐MSGs and WS‐MSGs was performed (Fig. 5B). It is worth noting that in the category of organismal systems, the immune system was the most represented pathway, and in the category of human diseases, viral infectious diseases were the most represented pathway. These findings indicated that the decrease in sericin 1 expression had a great impact on the cellular immune system, which was consistent with the results of the above transcriptomic analysis. The DEPs involved in the immune system and viral infectious disease pathways were then analyzed. The upregulated DEPs in HS‐MSGs included proteasome activator complex subunit 3 (PSME3), calreticulin, SCARB4, cuticular protein RR‐2 motif 96 (CPR96), CPR20, and some enzymes, while the significantly downregulated proteins included several cuticular proteins (CPR4, CPR5, CPR21, CPR46, CPR51, and CPR104) and some enzymes. PSME3 plays an important role in the regulation of the immune response [47] and interacts with c‐Myc [48] and nuclear factor kappa‐B (NF‐kB) [49]. The expression of *c‐Myc* was significantly downregulated in HS‐MSGs, and the expression of *inhibitor of nuclear factor kappa‐B kinase* was significantly upregulated in HS‐MSGs (Table S8). Calreticulin plays an important role in defense against infectious agents [41], and its gene expression was found to be significantly upregulated in the transcriptomic analysis (Table S8). SCARB4 plays an important role in innate immunity [42], and the expression of the *SCARB4* gene was found to be significantly upregulated in transcriptomic analysis (Table S8). Insect cuticular proteins are structural proteins that together with chitin form a cuticle barrier to resist the invasion of environmental pathogens and protect the insect from injury and dehydration [50]. Consistent with the similar functions of sericin 1 and cuticular proteins, many significantly differentially expressed cuticular proteins were identified, indicating a relatively direct link between sericin 1 and some cuticular proteins. These results confirmed that the decrease in sericin 1 expression significantly affected the cellular immune system and further suggested that sericin 1 may be directly involved in the cellular immune response.

Furthermore, the DEPs identified from the comparison of HS‐MSGs and WS‐MSGs were further screened according to the criteria of a fold change ≥ 1.5 and a *P* ≤ 0.05. After screening, 20 upregulated DEPs and 29 downregulated DEPs were obtained (Table S15). The 20 upregulated DEPs were classified into five categories according to functional annotation information: protease inhibitors (3); enzymes (7); cuticular proteins (1); other (4); and proteins of unknown functions (5). Notably, three protease inhibitors were significantly upregulated in HS‐MSGs (two zonadhesin proteins and one serine protease inhibitor), while no protease inhibitors were significantly downregulated. As described for the transcriptomic analysis above, the cells responded to the dramatic decrease in sericin 1 expression by increasing the expression of some protease inhibitors. These results further confirm that there is a relatively direct relationship between sericin 1 and some protease inhibitors that is associated with cellular immune responses.

## Discussion

In this study, CRISPR/Cas9‐mediated gene editing technology was successfully applied to target the third exon of the *sericin 1* gene. In the G3 generation, homozygous mutants with an 11‐bp insertion were obtained. This insertion caused a frameshift mutation and a premature stop at 323 aa. Sericin protein in silkworm cocoon silk is composed of mainly sericin 1 protein and a small amount of sericin 3 protein [11]. However, the phenotypic observation of cocoon shells showed that there were no significant differences between HS and WS individuals, for two possible reasons. First, *sericin 1* is capable of producing five kinds of mRNAs by alternative splicing, named ser1A, ser1A’, ser1B, ser1C, and ser1D, of which only ser1B and ser1C contain the third exon [51]. In HS‐MSGs, ser1A, ser1A’, and ser1D without the third exon can be translated normally and secreted into the cocoon shell with the silk fibroin protein. Second, the mutant *sericin 1* gene can be transcribed and produce abnormal sericin 1 protein under the action of an endogenous promoter. The abnormal sericin 1 protein may be able to replace part of the function of the normal sericin 1 protein and can be secreted into the cocoon shell with the silk fibroin protein. In addition, the phenotypic observation and weighing results of PSGs showed that there were no significant differences between HS and WS individuals, indicating that downregulation of *sericin 1* gene expression did not affect the expression of the silk fibroin gene. When silk fibroin protein passes through the MSG, it forms a double‐layer concentric cylinder with liquid silk fibroin inside and liquid sericin outside, and the two proteins are separated without mixing [52]. These results indicate that there is no associated regulatory pathway between sericin 1 and silk fibroin protein, which further explains the normal formation of HS cocoons.

Sericin was neglected for a very long time in the silk reeling industry because of the lack of recognition of sericin and the limitations of research. With the development of research, it has been found that sericin has many excellent biological activities, including antioxidant [53], anticoagulant [15], and bacteriostatic [13] activities and that it promotes cell adhesion and proliferation [17]. It is worth noting that sericin itself has the ability to resist microbial infection, which means that sericin 1, as the main component of sericin protein in the cocoon shell, may not only be a secretory protein but also play a role in the cellular immune system. In this study, transcriptomic analysis and proteomic analysis showed that both DEGs and DEPs identified from the comparison of HS‐MSGs and WS‐MSGs were significantly enriched in immune system‐related pathways, which indicated that *sericin 1* gene expression downregulation had a great impact on the cell immune response and suggests that sericin 1 may directly participate in cell immune defense. Further analysis of DEGs and DEPs involved in immune system‐related pathways revealed the presence of shared genes, including *calreticulin*, *SCARB4*, and others. The expression of *calreticulin* and *SCARB4* was significantly upregulated in HS‐MSGs at both the mRNA and protein levels, which further confirms the role of sericin 1 in the cellular immune response.

Protease inhibitors are highly abundant in silkworm cocoons and are considered to play an important role in resisting microbial infection [44]. The abundance of protease inhibitors in wild silkworm cocoons is significantly higher than that in domestic silkworm cocoons, further confirming the role of protease inhibitors in resistance to microbial infection given that wild silkworms are exposed to more environmental stress than domestic silkworms [45]. Several types of protease inhibitors have been reported in silkworm cocoons, including Kunitz, Kazal, TIL, AMFPI, ITI, etc., many of which have been shown to play significant roles in insect defense functions [44, 46, 54]. These results suggest that sericin 1 and protease inhibitors cooperate in the fight against microbial infection. In this study, 16 genes encoding protease inhibitors were significantly upregulated in HS‐MSGs compared to WS‐MSGs, suggesting that cells responded to the downregulation of sericin 1 expression by upregulating the expression of protease inhibitors to achieve stability of the immune system. Notably, all 16 protease inhibitors were detected in silkworm cocoons, suggesting that the secretion of protease inhibitors was strictly regulated rather than resulting from leakage from MSG cells. In addition, many differentially expressed cuticular proteins were identified from the comparison of HS‐MSGs and WS‐MSGs in proteomic analysis. Considering the similar functions between sericin 1 and cuticular proteins, it is inferred that there is a relatively direct regulatory pathway between sericin 1 and some cuticular proteins.

In summary, the results presented in this study reveal that downregulation of sericin 1 expression has a significant effect on the cellular immune system, suggesting that sericin 1 may be directly involved in the cellular immune response. In addition, the results reveal that there is a relatively direct and strict regulatory pathway between sericin 1 and some protease inhibitors and indicate that secretion of these proteins plays a synergistic role in the resistance to microbial infection in silkworm cocoons.

## Conflict of interest

The authors declare that there are no conflicts of interest.

## Author contributions

XY and BZ designed experiments. XY, SZ, MW, JR, XT, XW, and BZ conducted experiments. XY and BZ performed data analysis. XY wrote the paper. BZ revised the manuscript and coordinated the study. All authors read the manuscript before submission.

## Supporting information


**Table S1.** Primers used for mutant identification and preparation of sgRNA.

**Table S2.** Injection and mutation frequency.

**Table S3.** Data from RNA sequencing.

**Table S4.** Mapping to the reference silkworm genome.

**Table S5.** Mapping to the reference silkworm gene sequences.

**Table S6.** Genes identified by RNA sequencing.

**Table S7.** DEGs identified from the comparison of the ZS‐MSGs with the WS‐MSGs.

**Table S8.** DEGs identified from the comparison of the HS‐MSGs with the WS‐MSGs.

**Table S9.** DEGs identified from the comparison of the ZS‐MSGs with the HS‐MSGs.

**Table S10.** KEGG pathway enrichment analysis of the DEGs identified from the comparison of the HS‐MSGs with the WS‐MSGs.

**Table S11.** Proteins identified by proteomic analysis.

**Table S12.** DEPs identified from the comparison of the ZS‐MSGs with the WS‐MSGs.

**Table S13.** DEPs identified from the comparison of the HS‐MSGs with the WS‐MSGs.

**Table S14.** DEPs identified from the comparison of the ZS‐MSGs with the HS‐MSGs.

**Table S15.** DEPs obtained by further screening.

**Fig. S1.** Predicted amino acid sequences of wild‐type and mutant Sericin 1 proteins. The wild‐type sequence is encoded by all nine exons. The matching amino acid sequence is shown in red. The numbers on the right indicate the amino acid residue positions of the proteins.
**Fig. S2.** Gene Ontology analysis of the DEGs identified from the comparison of HS‐MSGs and WS‐MSGs. GO terms with corrected *p*‐values <= 0.05 were considered significantly enriched.
**Fig. S3.** Unique peptides for the identified proteins.
**Fig. S4.** Gene Ontology analysis of the DEPs identified from the comparison of HS‐MSGs and WS‐MSGs.

## Data Availability

All data generated or analyzed in this study are included in this manuscript [in the Supplementary Material]. Reads of transcriptome were deposited in the Sequence Read Archive (SRA) database of NCBI with the accession numbers PRJNA741673.
