# Supplementary material for: Role of sericin 1 in the immune system of silkworms revealed by transcriptomic and proteomic analyses after gene knockout
Source: FEBS Open Bio. 2021 Jul 13;11(8):2304–18. doi: 10.1002/2211-5463.13239 (PMC8329953; doi:10.1002/2211-5463.13239)
Supplement: Supplementary file 16 — Fig. S1. Predicted amino acid sequences of wild‐type and mutant Sericin 1 proteins. The wild‐type sequence is encoded by all nine exons. The matching amino acid sequence is shown in red. The numbers on the right indicate the amino acid residue positions of the proteins. Fig. S2. Gene Ontology analysis of the DEGs identified from the comparison of HS‐MSGs and WS‐MSGs. GO terms with corrected p‐values <= 0.05 were considered significantly enriched. Fig. S3. Unique peptides for the identified proteins. Fig. S4. Gene Ontology analysis of the DEPs identified from the comparison of HS‐MSGs and WS‐MSGs. [file FEB4-11-2304-s012.docx]

**Role of sericin 1 in the immune system of silkworms revealed by transcriptomic and proteomic analyses after gene knockout**

Xiaogang Ye^1†^, Shuo Zhao^1†^, Meiyu Wu^1^, Jinghua Ruan^1^, Xiaoli Tang^1^, Xiaoxiao Wang^1^ & Boxiong Zhong^1^*

^1^ College of Animal Sciences, Zhejiang University, Hangzhou, P. R. China

* Correspondence should be addressed to B. Z. ([bxzhong@zju.edu.cn](mailto:bxzhong@zju.edu.cn)). Tel.:/Fax: +86-571-86971302.

† Xiaogang Ye and Shuo Zhao contributed equally to this work.

| **List of all supporting components** | |
| --- | --- |
| Figure S1 | Predicted amino acid sequences of wild-type and mutant Sericin 1 proteins |
| Figure S2 | Gene Ontology analysis of the DEGs identified from the comparison of HS-MSGs and WS-MSGs |
| Figure S3 | Unique peptides for the identified proteins |
| Figure S4 | Gene Ontology analysis of the DEPs identified from the comparison of HS-MSGs and WS-MSGs |
| Table S1 | Primers used for mutant identification and preparation of sgRNA |
| Table S2 | Injection and mutation frequency |
| Table S3 | Data from RNA sequencing |
| Table S4 | Mapping to the reference silkworm genome |
| Table S5 | Mapping to the reference silkworm gene sequences |
| Table S6 | Genes identified by RNA sequencing |
| Table S7 | DEGs identified from the comparison of the ZS-MSGs with the WS-MSGs |
| Table S8 | DEGs identified from the comparison of the HS-MSGs with the WS-MSGs |
| Table S9 | DEGs identified from the comparison of the ZS-MSGs with the HS-MSGs |
| Table S10 | KEGG pathway enrichment analysis of the DEGs identified from the comparison of the HS-MSGs with the WS-MSGs |
| Table S11 | Proteins identified by proteomic analysis |
| Table S12 | DEPs identified from the comparison of the ZS-MSGs with the WS-MSGs |
| Table S13 | DEPs identified from the comparison of the HS-MSGs with the WS-MSGs |
| Table S14 | DEPs identified from the comparison of the ZS-MSGs with the HS-MSGs |
| Table S15 | DEPs obtained by further screening |


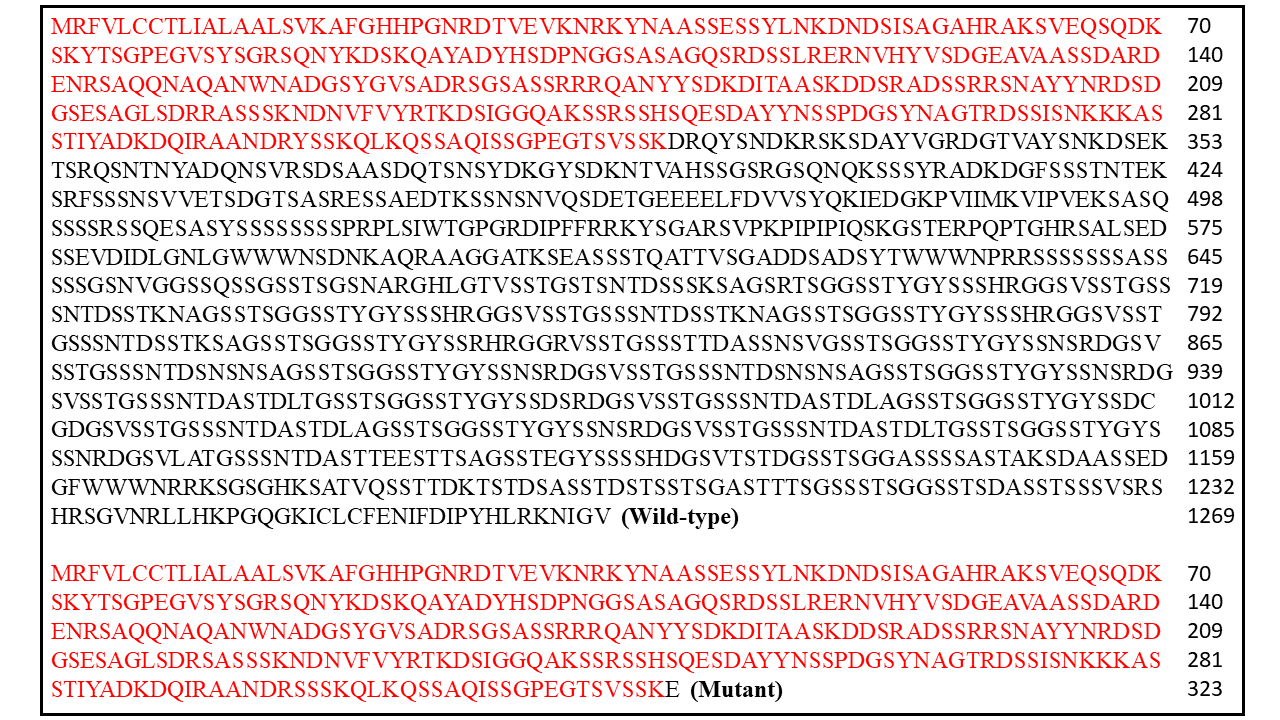


**Supplementary Figure S1**. Predicted amino acid sequences of wild-type and mutant Sericin 1 proteins. The wild-type sequence is encoded by all nine exons. The matching amino acid sequence is shown in red. The numbers on the right indicate the amino acid residue positions of the proteins.


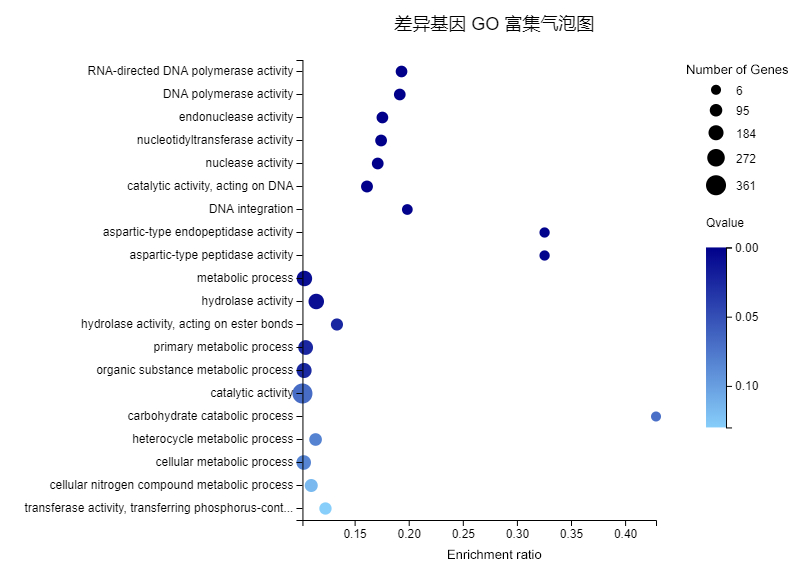


**Supplementary Figure S2**. Gene Ontology analysis of the DEGs identified from the comparison of HS-MSGs and WS-MSGs. GO terms with corrected *p*-values <= 0.05 were considered significantly enriched.


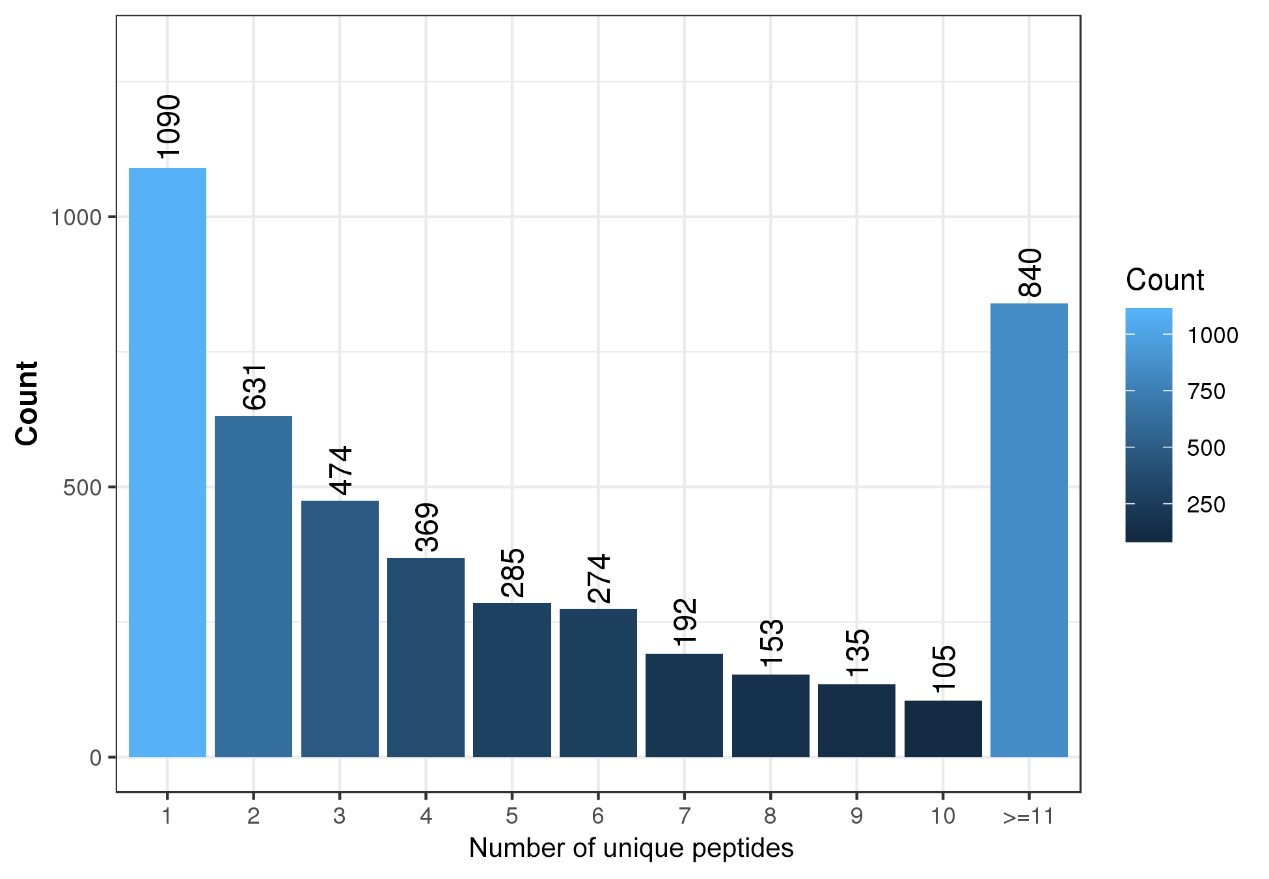


**Supplementary Figure S3**. Unique peptides for the identified proteins.


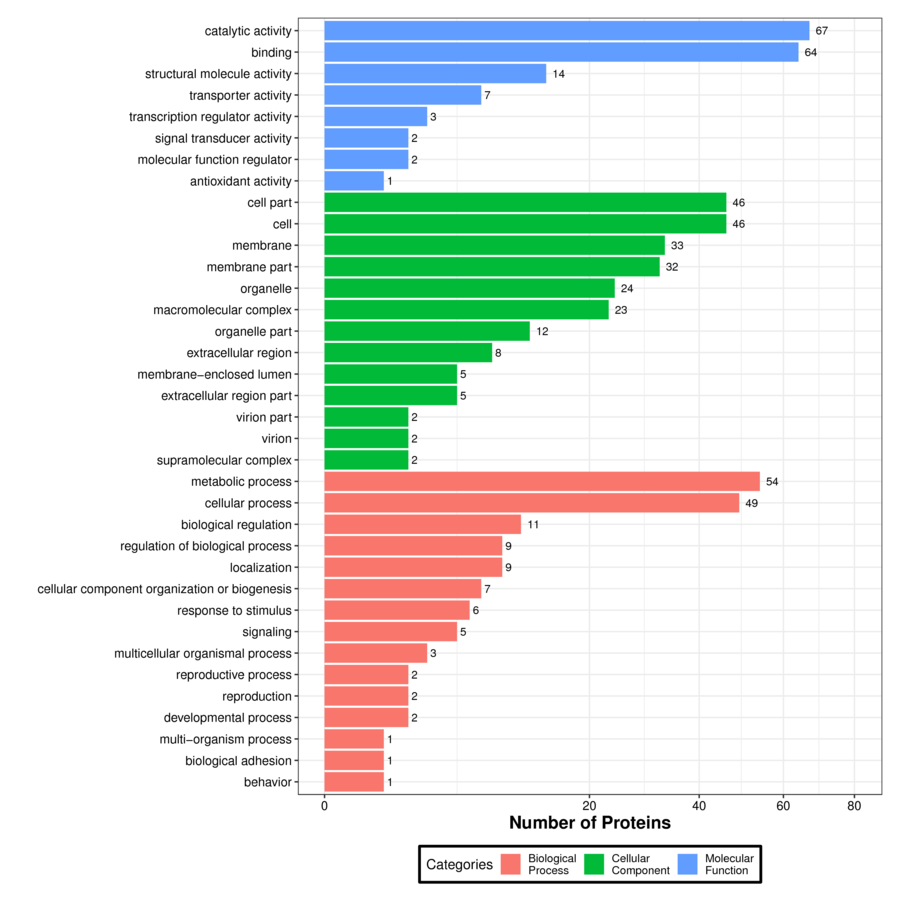


**Supplementary Figure S4**. Gene Ontology analysis of the DEPs identified from the comparison of HS-MSGs and WS-MSGs.
